# Anti-Mycobacterial Antibodies in Paired Cerebrospinal Fluid and Serum Samples from Japanese Patients with Multiple Sclerosis or Neuromyelitis Optica Spectrum Disorder

**DOI:** 10.3390/jcm7120522

**Published:** 2018-12-07

**Authors:** Kazumasa Yokoyama, Davide Cossu, Yasunobu Hoshino, Yuji Tomizawa, Eiichi Momotani, Nobutaka Hattori

**Affiliations:** 1 Department of Neurology, Juntendo University School of Medicine, Tokyo 113-8421, Japan; davide@juntendo.ac.jp (D.C.); yshoshi@juntendo.ac.jp (Y.H.); tmzw1977@gmail.com (Y.T.); nhattori@juntendo.ac.jp (N.H.); 2 Department of Treatment and Research in Multiple Sclerosis and Neuro-intractable disease, Juntendo University School of Medicine, Tokyo 113-8421, Japan; 3 Advanced Research Institute for Health Science, Juntendo University School of Medicine, Tokyo 113-8421, Japan; 4 Department of Human-care, Tohto College of Health Sciences, Saitama 366-0052, Japan; eiichimomotani@gmail.com

**Keywords:** Mycobacterium, Japan, humoral response, cerebrospinal fluid, multiple sclerosis, NMOSD, MBP

## Abstract

Local synthesis of antibodies and presence of oligoclonal bands in the cerebrospinal fluid (CSF) are hallmarks of multiple sclerosis (MS). We investigated the frequency of antibodies against mycobacterial and relevant human epitopes in the CSF of patients with MS or neuromyelitis optica spectrum disorder (NMOSD) and whether these antibodies differed from those present in the serum. Matched serum and CSF samples from 46 patients with MS, 42 patients with NMOSD, and 29 age-matched and sex-matched control subjects were screened retrospectively for the presence of antibodies against *Mycobacterium avium* subsp. *paratuberculosis* (MAP) pentapeptide (MAP_5p), MAP_2694_295–303_, and myelin basic protein (MBP)_85–98_ peptides by using indirect ELISA. Serum levels of anti-MAP_5p and anti-MAP_2694_295–303_ antibodies were highly prevalent in patients with MS when compared to patients with NMOSD and controls. Several patients with MS had detectable anti-MAP_5p and anti-MAP_2694_295–303_ antibodies in the CSF. Furthermore, a group of patients with MS showed intrathecally restricted production of antibodies against these peptides. Women appeared to mount a stronger humoral response to mycobacterial peptides than men. No significant difference in the frequency of anti-MBP_85–98_ antibodies was found between patients with MS and those with NMOSD. These data highlight the zoonotic potential of MAP, which suggests its involvement in MS etiopathogenesis.

## 1. Introduction

Although T cells directly mediate inflammatory damage within the central nervous system (CNS) in multiple sclerosis (MS), emerging evidence has highlighted a crucial role of B cells as precursors of antibody-secreting plasma cells and as antigen-presenting cells for T-cell activation [1]. While risk factors remain unknown, cumulative data suggest that microorganisms such as viruses and/or bacteria may play a fundamental role in MS pathogenesis [2]. 

Increased immunoglobulin G (IgG) intrathecal synthesis in the CNS is considered a hallmark of clinically defined MS [3]. Both elevated IgG index and oligoclonal bands (OCBs) are detectable in more than 90% of patients with MS [3]. The study of intrathecal synthesis is a quantitative and sensitive method for determining the presence of specific antibodies in the CNS and the Antibody Index (AI) is calculated to detect brain-derived microorganism-specific antibodies in the CSF [4]. 

Antibodies against different *Mycobacterium avium* subspecies *paratuberculosis* (MAP), Epstein–Barr virus (EBV), and human homologue peptides including myelin basic protein (MBP) have been detected in the cerebrospinal fluid (CSF) of Italian patients with MS during the relapse phase [5], which indicates a role of the bacterium or virus in enhancing inflammation through a molecular mimicry mechanism [6].

Seroprevalence studies have shown a stronger humoral response elicited by the MAP_2694 protein (UniProt accession no. Q73WG6) in Sardinian patients with MS when compared to healthy controls [7,8]. The screening of MS sera using a peptide library spanning the entire amino acid sequence of MAP_2694 protein identified an immunodominant epitope, MAP_2694_295–303_, located within a region showing a high homology to the T-cell receptor gamma-chain protein [9]. This peptide was shown to specifically bind to antibodies present in the sera of patients with relapsing remitting MS (RRMS) but not to those of healthy subjects. The specificity of this binding was proven by competitive assays [9]. The in silico molecular modeling study demonstrated that the MAP_2694_295–303_ peptide displays a binding affinity to MS-associated HLA-DR molecules [10]. Furthermore, a recent article has revealed high serum levels of antibodies against the MAP_2694_295–303_ peptide in Japanese patients with MS (12 RRMS, 2 secondary progressive, and 1 primary progressive) and those with a clinically isolated syndrome (CIS) [11]. 

Since none of these retrospective studies has determined whether these antibodies were also present in the CSF and whether they were intrathecal or blood-derived, the first objective of this study was to investigate potentially specific intrathecal MAP_2694_295–303_ IgG synthesis in patients with MS, patients with neuromyelitis optica spectrum disorder (NMOSD), and disease control subjects. 

In order to demonstrate the specificity of the antibody response to MAP, the second objective of our work was to perform an antibody screening against MAP pentapeptide (MAP_5p) in all samples. The synthetic peptide MAP_5p is a variant of lipopentapetide (L5P) without an N-terminally C_20_ saturated fatty acid for a higher antibody affinity [12]. L5P is a cell-wall component able to discriminate MAP from other non-tuberculosis pathogenic mycobacteria [12]. Anti-L5P antibodies have been detected not only in MAP infected animals [12] but also in patients with Crohn’s disease and with Type 1 diabetes [13]. 

Furthermore, human MBP is an important candidate autoantigen in MS and the region spanning the amino acids 85–98 has been identified as an immunodominant MBP peptide [14]. Increased frequencies of antibodies to MBP_85–98_ have been detected in the serum and CSF of patients with RRMS compared with controls [5,15]. Inhibition assays revealed that serum antibodies recognizing MBP_85–98_ cross-reacted with a homologous peptide belonging to a MAP_0106c protein likely through a molecular mimicry mechanism [15]. Hence, the third objective of our study was to quantify the frequency of antibodies against myelin basic protein (MBP)_85–98_.

Lastly, we investigated a potential link between detected CSF antibodies and unique clinical MS features in order to elucidate a role exerted by these peptides in the CNS.

## 2. Materials and Methods

### 2.1. Patients

A total of 117 paired serum and CSF samples were obtained from Japanese patients newly recruited at Juntendo University School of Medicine, Tokyo, Japan. The study protocol was approved by the National Ethical Committee of the Juntendo University School of Medicine (Approval No. 205). All the methods were conducted in “accordance” with the approved guidelines. All subjects provided written informed consent prior to participation. 

The study included 46 patients with MS (women/men = 35/11, mean age 38.5 ± 10.3) diagnosed according to McDonald criteria [16]. The clinical characteristics of patients with MS were as follows: 36 RRMS, 2 secondary progressive, 1 primary progressive, 5 diagnosed with CIS, and 2 with radiologically isolated syndrome (RIS). The duration of the disease was 6.9 ± 6 years and the mean age at MS onset was 31.3 ± 7.9 years. The expanded disability status scale (EDSS) of these patients was a mean of 2 and ranged from 0 to 7. The EDSS scale ranges from 0 to 10 in 0.5 unit increments that represent increasingly higher levels of disability. Scoring is based on an examination by a neurologist.

In addition, 42 patients diagnosed with NMOSD (women/men = 29/13, mean age 50.1 ± 17.2) who fulfilled the 2015 international consensus diagnostic criteria were also enrolled in the study [17]. The NMOSD group included patients with recurrent optic neuritis and/or longitudinally extensive transverse myelitis alone including aquaporin-4 (AQP4)-positive and AQP4-negative. The duration of the disease was 7.5 ± 8 years and the mean age at onset was 42.3 ± 15.1 years.

The disease control group comprised 29 subjects matched for age and sex (women/men = 22/7, mean age 48.6 ± 17.1) from the Juntendo University’s database with different neurologic pathologies (Guillain–Barré syndrome (*n* = 4), acute disseminated encephalomyelitis (*n* = 3), multiple cranial neuritis (*n* = 2), neurosarcoidosis (*n* = 2), encephalitis (*n* = 2), acute cerebellitis (*n* = 1), HTLV-1 associated myelopathy (*n* = 1), Tolosa–Hunt syndrome (*n* = 1)), psychiatric pathologies (dementia (*n* = 2), schizophrenia (*n* = 1)), and without specific neurological disorders at the time of magnetic resonance imaging (MRI) (headache (*n* = 2), white-matter abnormalities (*n* = 5), myasthenia gravis (*n* = 2), and Sjögren syndrome (*n* = 1)).

### 2.2. Serum and CSF Samples

Three patients with MS were treated with interferon-beta for at least 6 months before the sample collection while 2 patients with NMOSD had received pulse steroids therapy in the 30 days prior to testing. All the other subjects were free from any treatment at the time of blood sampling and lumbar puncture.

All CSF and paired serum samples were routinely analyzed for IgG, IgM, IgA, AQP4, albumin, and OCBs.

Two milliliters of the blood sample were collected from each subject and mononuclear cells were then isolated (within the 3 h following collection) using density gradient centrifugation with Ficoll-Paque media (Sigma-Aldrich, St. Louis, MO, USA). Immediately after centrifugation, sera were stored at −80 °C until use.

Two milliliters of CSF were collected from each subject through a lumbar puncture according to standard procedures. Samples were centrifuged (2000× *g*/10 min) to remove cells and debris within the 30 min following collection before being aliquoted at −80 °C until analysis.

### 2.3. Antigens

Synthetic MAP_5p (DPhe-NMeVa1-Ile-Phe-Ala-OMe) was synthesized at >95% purity (GenScript, Piscataway, NJ, USA) while MAP_2694_295–303_ (ADVTIADPT) and MBP_85–98_ (ENPVVNFFKNIVTP) peptides were synthesized at >90% purity (GenScript, Piscataway, NJ, USA). All peptides were stored in single-use aliquots at −80 °C. 

### 2.4. Enzyme-Linked Immunosorbent Assay

Nunc-immuno-MicroWell-96 well solid plates (Thermo Fisher Scientific, Waltham, MA, USA) were coated with 50 µL/well of each peptide and diluted in ELISA coating buffer (Bio-Rad, Tokyo, Japan) at a final concentration of 10 μg/mL overnight at +4 °C. The plates were saturated with Blocking One (Nakalai Tesque, Kyoto, Japan) buffer for 1 h at room temperature (+25 °C). After rinsing with PBS-T (10 mM PBS, pH 7.0, containing 0.5% Tween 80) four times, serum samples were added to the plates (1:100 in Blocking One) and incubated for 2 h at room temperature. CSF samples were added undiluted. After repeated washing, the plates were incubated with 100 µL/well of horseradish peroxidase-labeled-goat anti-human IgG polyclonal Ab (Southern Biotech Associates, Inc., Birmingham, AL, USA) for 1 h at room temperature. After washing, the wells were incubated with 100 µL/well of ABTS Peroxidase System (SeraCare Life Sciences, KPL, Gaithersburg, MD, USA) for 10 min in the dark at room temperature. The optical density was read at 650 nm in a Benchmark Plus Microplate Reader (Bio-Rad, Tokyo, Japan). All serum and CSF samples were assayed in duplicates. The background, which was determined by incubating immobilized peptides with a secondary antibody alone, was subtracted from each measurement. The cut off for positivity in each assay was calculated by ROC analysis and set at 95% specificity. The sensitivity was chosen accordingly. The results were normalized to a positive control serum included in all experiments.

### 2.5. Determination of Intrathecal IgG Synthesis

The intrathecal synthesis of MAP_2694_295–303_, MAP_5p, and MBP_85–98_ specific antibodies was determined using the AI and calculated as AI = *Q*_spec_/*Q*_total_ [18]. *Q*_spec_ is the CSF/serum quotient of specific antibodies while *Q*_total_ is the CSF/serum quotient of the total IgG. In the case where *Q*_total_ > *Q*_Lim_, AI = *Q*_spec_/*Q*_Lim_, according to Formula [18]: *Q*_Lim_ (IgG) = $0.93\sqrt{{\left(QAlb\right)}^{2}+6\;\times \;10^{-6}}\;-1.7\times 10^{3}$.

Values of AI >1.5 indicate specific local antibody synthesis in the CNS.

### 2.6. Magnetic Resonance Imaging (MRI)

The MRI was performed using a 3T magnetic resonance scanner (Achieva, Philips Medical Systems, Best, and The Netherlands) at Juntendo Hospital, as previously reported [19]. 

### 2.7. Visual Evoked Potential (VEP)

The visual stimulation protocol consisted of a full-field stimulation of each eye with a checkerboard patterns fixation point. The VEP parameters recorded were P100 wave latency (normal mean, 98.9 ms, upper limit, 110.0 ms). Patients with a P100 latency value greater than 110.0 ms were considered abnormal [19].

### 2.8. Statistical Analysis

The Fisher exact χ^2^ test was used to test the differences between non-parametric variables. The comparisons between patients with MS or NMOSD and disease control subject IgG serum titers were performed using the Mann–Whitney *U* test. Student’s *T* test was used to examine the distribution of antibodies titers between females and males. *p* values < 0.05 were considered significant. The area under the receiver operating characteristic curve (AUC) was calculated to determine the performance of ELISA in discriminating MS from control patients. The optimal cut-off value was determined by using ROC analysis, as previously reported [11]. Correlations were investigated by linear regression. All statistical analyses were performed using Prism 6.0 software (GraphPad, San Diego, CA, USA).

## 3. Results

### 3.1. Various Specific IgG Antibodies are Present in Paired Serum and CSF Samples of Patients with MS or NMOSD

**Figure 1:**
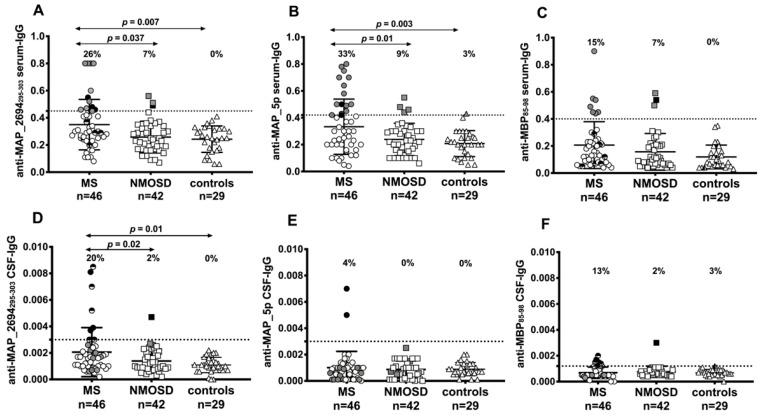
Detection rates for MAP_2694_295–303_, MAP_5p, and MBP_85–98_ IgG antibodies in matched serum (**A**–**C**) and cerebrospinal fluid (CSF) (**D**–**F**) samples by using indirect ELISA. The percent fraction of antibody-positive sera is indicated on the top of each distribution. Black symbols represent patients with antibodies in both serum and CSF while grey and half-black symbols represent patients with specific antibodies only in the serum or in the CSF, respectively. The horizontal bars represent the mean with a standard deviation while dashed lines indicated cut-off values for positivity calculated by ROC analysis. The *p*-values, significant if <0.05, are indicated by two-headed arrows.

Figure 1A shows the distribution of anti-MAP_2694_295–303_ antibodies in the serum of MS, NMOSD, and disease control groups. Twelve out of 46 (21%) patients with MS, 3 out of 42 (7%) patients with NMOSD, and none of the disease control subjects were positive for antibodies against MAP_2694_295–303_ (*p* = 0.037 and *p* = 0.007, respectively)_._ Among antibody-positive patients with MS, all untreated patients, and those in the stable phase, 10 had RRMS, 1 had primary progressive MS, and 1 was diagnosed with RIS. Among the 3 antibody-positive patients with NMOSD, 2 were AQP4-negative and 1 was AQP4-positive.

Measurements of the anti-myelin oligodendrocyte glycoprotein (MOG) antibody, which is another biomarker candidate antibody for CNS demyelinating disorders, were not performed in these patients.

**Figure 2:**
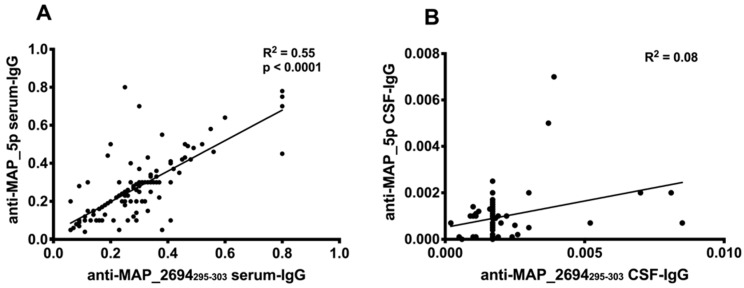
Correlations between antibodies recognizing MAP_2694_295–303_ and MAP_5p antigens. The distributions represent correlation between antibodies against the mycobacterial peptides detected in the serum (**A**) or CSF (**B**) of all 116 study subjects. Each circle represents the titer for one patient.

Anti-MAP_5p antibodies were detected in 15 (33%) patients with MS, 4 (9%) patients with NMOSD, and in 1 disease control patient with white-matter abnormalities (*p* = 0.01 and *p* = 0.003, respectively) (Figure 1B). A strong correlation between titers of antibodies directed against MAP_5p and MAP_2694_295–303_ was observed among all subjects (Figure 2A). All 12 anti-MAP_2694_295–303_ positive patients with MS also displayed a strong humoral response to MAP_5p antigen (R^2^ = 0.55, *p* < 0.0001) with a coincidence of positivity of 91% in MS subjects.

Among the 4 antibody-positive patients with NMOSD, 2 were AQP4-negative and 2 were AQP4-positive.

Although the MBP_85–98_ peptide was recognized by 6 patients with RRMS and 3 with NMOSD that were AQP4-antibody positive (Figure 1C), there was no significant difference between groups. Furthermore, 3 out of 6 patients with RRMS were double positive for anti-MAP_2694_295–303_ and anti-MBP_85–98_ antibodies while only 1 patient with NMOSD (AQP4-negative, OCB-negative) showed antibodies against both peptides. 

Concerning the antibody frequency in the CSF, 9 patients with MS (7 RRMS, 1 primary progressive, and 1 CIS) and 1 with NMOSD (both AQP4-positive and OCB-positive) had detectable anti-MAP_2694_295–303_ antibodies (*p* = 0.02) (Figure 1D) while 2 patients with MS (1 RRMS and 1 primary progressive) were positive to MAP_5p peptide (Figure 1E). There was no correlation between the CSF anti-MAP_5p antibody and the CSF anti-MAP_2694_295–303_ antibody levels among all patients (R^2^ = 0.08) (Figure 2B). No significant difference regarding CSF MBP_85–98_ antibody frequency was found between MS (5 RRMS patients) and NMOSD (1 patient AQP4-negative, OCB-negative) (Figure 1D).

### 3.2. Synthesis of Intrathecal Anti-Mycobacterial Antibodies in Patient with MS

Antibodies can either access the brain through circulation when the blood–brain barrier integrity is compromised or be produced locally within the CNS. To discriminate between blood-derived and locally-synthetized antibodies, the AI for each antigen was calculated.

**Figure 3:**
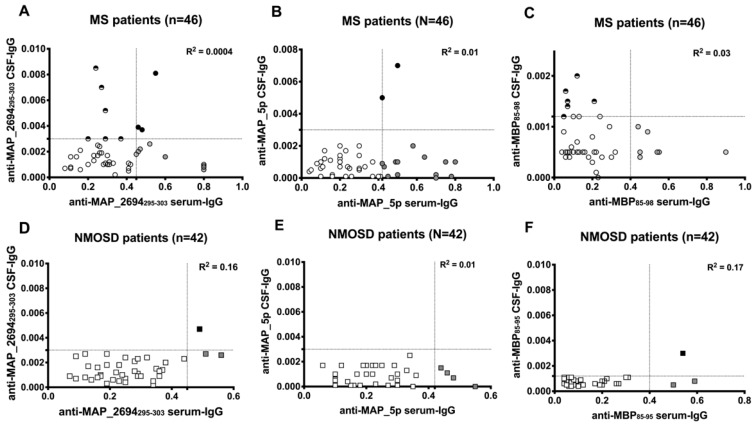
Correlations between CSF and serum anti-MAP_2694_295–303_, anti-MAP_5p, and anti-MBP_85–95_ antibody titers from the patients with multiple sclerosis (**A**–**C**) and NMOSD (**D**–**F**). Each circle represents the titer for one patient. Dotted lines indicate the cut-off for positivity used in each assay, as calculated by ROC analysis.

Eight out of 9 patients with MS showing detectable anti-MAP_2694_295–303_ antibodies in the CSF had intrathecal antibody production (AI >1.5) against the MAP_2694_295–303_ peptide. All patients had been free from therapy for at least 6 months before sampling and none of them presented blood-brain barrier damage (albumin quotient < 7 × 10^−3^). Figure 3A shows an absence of correlation between serum and CSF anti-MAP_2694_295–303_ antibody titers. Two patients (RR and primary progressive MS) also showed intrathecal IgG synthesis against MAP_5p peptide (Figure 3B).

Conversely, the patient with NMOSD showing detectable anti-MAP_2694_295–303_ antibodies in the CSF presented altered blood-brain barrier permeability (elevated albumin quotient = 9 × 10^−3^) and anti-MAP_2694_295–303_ antibodies were detected in both serum and CSF (AI < 1.5) at the same time of sampling (Figure 3D), which suggests non-specific intrathecal synthesis or passive diffusion from peripheral blood circulation.

All anti-MBP_85–98_ antibodies detected in the CFS of patients with MS or NMOSD were locally synthesized in the CNS (AI >1.5) (Figure 3C,F), but no significant difference was found between the groups in the frequency of anti-MBP_85–98_ antibodies or albumin quotients (Table 1).

### 3.3. Correlation Between Antibody Positivity and Clinical Characteristics of Patients with MS

CSF parameters of 117 subjects are reported in Table 1. Male patients with MS did not mount a humoral immune response against mycobacterial peptides, which suggests differences in the immune response to bacteria between women and men. The frequencies of anti-MAP_2694_295–303_ and anti-MAP_5p serum titers was significantly higher in the women compared to the men (*p* = 0.02 and *p* = 0.03, respectively). The increase of MS among women in some population might be caused by different factors like sex hormones or by a different genetic susceptibility to pathogens [20].

All patients with MS or NMOSD showing detectable anti-MAP_2694_295–303_ and anti-MAP_5p antibodies in the CSF had a high IgG index and were OCB-positive. Baseline EDSS scores were higher in the CSF of anti-mycobacterial positive patients (3.9 ± 1.7) in comparison with antibody-negative patients (1.5 ± 0.9. *p* < 0.0001). 

Concerning the frequency of magnetic resonance imaging (MRI) abnormalities between patients with MS with and without mycobacterial antibodies, all MAP-positive subjects with MS showed demyelinating lesions in the cerebral cortex and subcortical areas. The lesions in the brain stem and optic nerve as well as lesions in the spinal cord were observed in 8 subjects. Relevant atrophy in the cerebellar cortex and in the upper cervical spinal cord was seen only in the primary progressive patient while 8 patients with RRMS presented mild brain atrophy. To date, 3 patients (1 RRMS, 1 primary progressive, and 1 secondary progressive) with AI > 1.5 with double positivity for MAP_2694_295–303_ and MAP_5p peptides presented cerebellar hemispheric lesions whose frequency and distribution are very low in Japanese patients [21] when compared to those of the classical type of MS in Caucasians.

No correlation was found with MBP_85–98_ and AQP4 antibodies between MAP-seronegative and seropositive patients. 

## 4. Discussion

Although CSF B cells and antibodies from patients with MS [22] have been reported to react to several myelin proteins such as MBP, MOG, and proteolipid protein, the antigens and the targets of the humoral immune response in the CNS are still unknown. In the present study, we used ELISA and cell-based assays to analyze humoral responses against different human and mycobacterial antigens in paired serum and CSF samples from patients with MS, patients with NMOSD, and control subjects as well as to investigate the intrathecal antibody production.

In our report, very few antibodies in the serum and CSF samples reacted with the MBP_85–98_ peptide and the difference between MS and NMOSD groups was not significant. We performed antibody screening with a selected single epitope located within the surface-exposed region of the MBP protein [14] rather than with the entire protein, which could eventually explain the low reactivity observed.

On the contrary, we detected a stronger humoral response against MAP_2694_295–303_ and MAP_5p peptides in the serum of patients with MS compared to those with NMOSD and the control group as well as a specific intrathecal synthesis of anti-MAP_2694_295–303_ and anti-MAP_5p IgG in the CSF of 8 and 2 patients with MS, respectively.

Our findings are in line with those obtained recently by Mameli et al. who observed an intrathecal specific synthesis (AI > 1.5) of IgG against various MAP peptides in Italian patients with MS [5]. This study differed from ours in that it evaluated humoral responses against MAP antigens that mimic homologue epitopes of EBV proteins encoded at different stages of viral replication [5]. We specifically focused on the humoral immune response toward a peptide from a MAP protein previously suggested to be related to MS [7,8,9,10] studying for the first time the intrathecal synthesis of specific antibodies. In addition, we detected a significant immune response to MAP_5p in all MAP_2694_295–303_ positive subjects, which confirms the specificity of a humoral response against cell wall components of MAP. Regarding the latter point, recent Japanese studies have shown a strong serum antibody response against MAP antigens in patients with MS [11,23], which highlights that indirect transmission of bacterial components following consumption of contaminated dairy products may have contributed to a recent increase in the number of individuals affected by MS in Japan [24].

The immune response during early disease phases is determined by a patient’s genetic predisposition especially following exposure to environmental factors that produce autoimmune dysregulation and activation of specific T cells [25]. Despite antibodies against protein elements of MAP were also found in several autoimmune diseases [13,24], MAP_2694_295–303_ antibodies seem to occur largely independent of other autoimmune disorders without involvement of the CNS, which highlights the actual importance of MAP_2694_295–303_ homology with the presence of intrathecal antibodies. The MAP_2694_295–303_ peptide possesses a high antigenic potential given its sequence homology with the constant region of the human gamma delta T-cell receptor [7,9]. Clonally expanded gamma delta T cells have been found in acute brain lesions, peripheral blood, and the CSF of patients with a disease onset of MS and they seem to regulate CNS inflammation [26]. In our experiments, we were not able to analyze the CSF gamma delta T cells due to a limited amount of samples available.

When individuals are exposed to mycobacterial antigens in the gut, molecular mimicry, epitope spreading, or bystander activation can occur and these events can trigger a breakdown in self-tolerance, which leads to an autoimmune response [27]. We speculate that antibodies raised against the MAP_2694_295–303_ peptide might cross-react through molecular mimicry with intraepithelial gamma delta T cells in the intestinal mucosa [28], which causes a partial depletion of these T-cell subsets and renders them unable to regulate the disease.

Ectopic lymphoid follicles present in the meninges of patients with secondary progressive MS are tertiary lymphoid organs where affinity maturation of B cells involved in CNS humoral autoimmunity occurs [29]. Additionally, new research also suggests that long-lived plasma cells can persist in the CNS and contribute to the chronic inflammation through the secretion of autoantibodies [30]. These data provide evidence that, while B cells initially encounter the antigen in the peripheral lymphoid organs, affinity maturation can occur both in peripheral and CNS tertiary lymphoid structures. MAP_2694_295–303_ antibodies might trigger and amplify an encephalitogenic immune response in the periphery by causing new waves of inflammatory CNS infiltration.

The simultaneous identification of intrathecal antibodies produced by patients with MS against non-specific antigenic epitopes from brain tissue (i.e., MAP_2694_295–303_) indicates antibody production by autoreactive plasma blasts or plasma cells. In a recent article, intrathecal inflammation was detected in post-mortem MS brains and CSF of patients with elevated meningeal inflammation and gray matter demyelination [31]. These results corroborate our findings of elevated MAP_2694_295–303_ IgG in CSF of subjects with a more active disease. Moreover, intrathecal synthesis of mycobacterial antibodies defined a small subgroup of patients with distinct clinical features that predominantly manifest cortical atrophy including cerebellar lesions.

Altogether, our data support the zoonotic potential of MAP and its involvement in the immunopathogenesis of MS. However, this is an association study and further research is needed to demonstrate a direct role of MAP_2694_295–303_ antibodies as an inflammatory and/or autoimmune component of the disease pathogenesis. As an animal model of MS, experimental autoimmune encephalomyelitis would allow us to address the encephalitogenic effect of MAP antigenic components. 

## Figures and Tables

**Table 1:** Cerebrospinal fluid (CSF) parameters.

CSF Parameters	MS (*n* = 46)	NMOSD (*n* = 42)	Controls (*n* = 29)	*p*
Oligoclonal IgG band positive	34 (74%)	11(26%)	7 (24%)	<0.0001 * <0.0001 **
IgG index ≥0.7 (%)	33 (72%)	7 (17%)	8 (27%)	<0.0001 * 0.0003 **
Albumin-Quotient (*Q*_Alb_) ≥ 7 × 10^−3^ (%)	10 (22%)	17 (40%)	6 (21%)	NS NS
AI-IgG MAP_2694_295–303_ >1.5 (mean)	8 (4.9)	0	0	0.006 * 0.02 **
AI-IgG MAP_5p >1.5 (mean)	2 (2.8)	0	0	NS NS
AI-IgG MBP_85–98_ >1.5 (mean)	5 (5.7)	1 (1.5)	0	NS NS

AI: antibody index frequency; MS: multiple sclerosis; NMOSD: neuromyelitis optica spectrum disorder; NS: not significant; * Statistically significant differences between MS and NMOSD or ** between MS and disease controls.
